# Prolonged Expression of a Putative Invertase Inhibitor in Micropylar Endosperm Suppressed Embryo Growth in Arabidopsis

**DOI:** 10.3389/fpls.2018.00061

**Published:** 2018-01-30

**Authors:** Bongeka Zuma, Mason B. Dana, Dongfang Wang

**Affiliations:** Department of Biology, Spelman College, Atlanta, GA, United States

**Keywords:** invertase inhibitor, sugar, embryo, endosperm, seed development, PRC2

## Abstract

Proper seed development requires coordinated growth among the three genetically distinct components, the embryo, the endosperm, and the seed coat. In Arabidopsis, embryo growth rate accelerates after endosperm cellularization, which requires a chromatin-remodeling complex, the FIS2-Polycomb Repressive Complex 2 (PRC2). After cellularization, the endosperm ceases to grow and is eventually absorbed by the embryo. This sequential growth pattern displayed by the endosperm and the embryo suggests a possibility that the supply of sugar might be shifted from the endosperm to the embryo upon endosperm cellularization. Since invertases and invertase inhibitors play an important role in sugar partition, we investigated their expression pattern during early stages of seed development in Arabidopsis. Two putative invertase inhibitors (*InvINH1* and *InvINH2*) were identified as being preferentially expressed in the micropylar endosperm that surrounds the embryo. After endosperm cellularization, *InvINH1* and *InvINH2* were down-regulated in a FIS2-dependent manner. We hypothesized that FIS2-PRC2 complex either directly or indirectly represses *InvINH1* and *InvINH2* to increase invertase activity around the embryo, making more hexose available to support the accelerated embryo growth after endosperm cellularization. In support of our hypothesis, embryo growth was delayed in transgenic lines that ectopically expressed InvINH1 in the cellularized endosperm. Our data suggested a novel mechanism for the FIS2-PRC2 complex to control embryo growth rate via the regulation of invertase activity in the endosperm.

## Introduction

Angiosperm seed is the product of double fertilization (Friedman, 1998; Linkies et al., 2010). During this process, one sperm cell fuses with the egg cell to produce the embryo, while the other sperm cell fuses with the central cell to produce the endosperm (Berger et al., 2008). After fertilization, the ovule integument develops to form the seed coat (Schneitz et al., 1995; Debeaujon et al., 2007). Therefore, seed development involves coordinated growth of three distinct organs: the diploid zygotic embryo, the triploid zygotic endosperm, and the diploid maternal seed coat (Garcia et al., 2005; Yang et al., 2008; Ingram, 2010). Most angiosperms, including Arabidopsis, have nuclear endosperm (Olsen, 2004; Bhojwani, 2009). During the initial phase of nuclear endosperm development, the nuclei divide rapidly without cellularization, forming a syncytium (Boisnard-Lorig et al., 2001). The syncytial phase is followed by endosperm cellularization, which coincides with the transition in the embryo from morphogenesis phase to growth phase (Goldberg et al., 1994; Olsen, 2004; Hehenberger et al., 2012). In addition to coordinated transition in development, the embryo and the endosperm also exhibit coordinated changes in growth rate. In eudicots with transient endosperm, embryo growth accelerates after the transition, while the endosperm stops growing soon after the transition and is eventually absorbed by the expanding embryo (Goldberg et al., 1994; Olsen, 2004; Baud et al., 2008; Hehenberger et al., 2012). This sequential growth pattern suggests that nutrient supplies are shifted from the endosperm to the embryo after endosperm cellularization.

The developmental transition in the endosperm is likely responsible for the acceleration in embryo growth rate after endosperm cellularization. Works in both Arabidopsis and rice have demonstrated that a chromatin-remodeling complex produced in the endosperm, the Polycomb Repressive Complex 2 (PRC2), is required for endosperm cellularization and the acceleration in embryo growth rate (Kiyosue et al., 1999; Sørensen et al., 2001; Folsom et al., 2014). In *PRC2* mutants, such as *mea, fis2, fie*, and *msi1*, the endosperm fails to cellularize and continues to proliferate, while the embryo fails to transition into the growth phase and aborts at heart stage (Ohad et al., 1996; Chaudhury et al., 1997; Kiyosue et al., 1999; Köhler et al., 2003). The PRC2 complex regulates many developmental processes by methylating histone H3 lysine 27 (H3K27me) to initiate gene silencing (Schubert et al., 2005; Schuettengruber et al., 2007; Zheng and Chen, 2011; Holec and Berger, 2012). These data indicate that PRC2-mediated gene silencing in the endosperm is involved in the regulation of nutrient allocation from the endosperm to the embryo.

Invertase plays an important role in sugar allocation during seed development. Since there is no symplastic connection between the seed coat, the endosperm, and the embryo (Stadler et al., 2005), active transport is required to move nutrients from maternal tissues to the endosperm at the chalazal interface, and from the endosperm to the embryo at the micropylar interface (Sanders et al., 2009; Pommerrenig et al., 2013; Chen et al., 2015; Sosso et al., 2015). At the chalazal interface, sucrose is unloaded from the phloem to apoplastic space, hydrolyzed by cell-wall-bound invertase to glucose and fructose, and subsequently imported into the endosperm (Cheng et al., 1996; Cheng and Chourey, 1999). Sucrose hydrolysis generates the concentration gradient that facilitates sucrose unloading from the seed coat to the endosperm at the chalazal interface (Weber et al., 1996; Sherson et al., 2003). However, it is not clear whether invertase is a part of the sugar transport mechanism at the micropylar interface.

There are two types of invertase: the acid invertase in the vacuole or the cell wall, and the neutral/alkaline invertase in the cytoplasm (Sturm, 1999). Since the acid invertases are relatively stable, their activity is mainly regulated by small proteinous invertase inhibitors (InvINHs) (Ruan et al., 2010). *InvINHs* and pectin methylesterase inhibitors (PMEIs) belong to the same superfamily that are characterized by four conserved cysteine residues (Camardella et al., 2000). To investigate whether invertases and InvINHs are involved in sugar transport across the micropylar interface, we analyzed the spatial and temporal expression pattern of invertase and the members of *InvINH/PMEI* superfamily during seed development. We identified two putative *InvINHs* (*InvINH1* and *InvINH2*) that were specifically expressed in the micropylar endosperm. Moreover, both genes were silenced by FIS2-PRC2 complex after endosperm cellularization. Finally, ectopic expression data suggested that *InvINH1* inhibited embryo growth.

## Materials and Methods

### Plant Materials and Growth Conditions

*Arabidopsis thaliana* ecotype Col-0 and *fis2-8* mutant (Wang et al., 2006) plants were housed in a walk-in Environmental Room (Norlake Scientific, Hudson, WI, United States) at 22°C under 16-h light/8-h dark long-day condition. Seeds were stratified for 4 days at 4°C before germination. Plants were grown in Pro-Mix BX soil (Premier Horticulture, Quakertown, PA, United States) and fertilized with Peters 20-20-20 (Scotts-Sierra Horticultural Products Company, Marysville, OH, United States).

### Microarray Data Analysis

Microarray data was downloaded from Gene Expression Omnibus database (accession no. GSE12404). The expression values were normalized with the GeneChip Robust Multiarray Averaging method (GC-RMA) implemented as a Bioconductor package under the R platform^1^ (Wu et al., 2004). Hierarchical cluster analysis (average linkage and Euclidean distance as similarity measure) was performed using Cluster 3.0 on log2-transformed expression values (de Hoon et al., 2004). The result of cluster analysis was visualized using Java TreeView (Saldanha, 2004).

The gene IDs for eight acid invertases (Glycoside Hydrolase Family 32) and nine neutral/alkaline invertases (Glycoside Hydrolase Family 100) were obtained from Carbohydrate-Active enZYmes Database^2^ (Sturm et al., 1999; Lammens et al., 2009). The gene IDs for 125 members of the plant InvINH/PMEI superfamily were obtained from SUPERFAMILY database^3^ (Wilson et al., 2009). Cluster analysis was conducted on 107 genes that are present on Affymetrix Arabidopsis ATH1 Genome Array, including 17 invertase genes and 90 InvINH/PMEI genes.

### Bioinformatics Analyses

The following online programs were used to predict the subcellular localizations of InvINH1 and InvINH2: PSORT^4^ (Nakai and Kanehisa, 1991), MultiLoc2^5^ (Blum et al., 2009), and YLoc^6^ (Briesemeister et al., 2010). All three programs were run with the default setting for plant proteins.

Genevestigator^7^ (Hruz et al., 2008) was used to analyze the expression pattern of *InvINH1* and *InvINH2*. The data selection includes a compendium of 5,825 wild-type *A. thaliana* samples profiled on the Affymetrix Arabidopsis ATH1 Genome Array platform. The Anatomy tool and Perturbations tool from the CONDITION SEARCH toolset were used to analyze the expression level of *InvINH1* and *InvINH2*, which are represented by the same Affymetrix probe (248823_s_at).

### RNA Isolation and Quantitative RT-PCR

Total RNAs were extracted from roots, stems, rosette leaves, closed floral buds from stage 0 to 12 (Smyth et al., 1990), and young siliques at 3 and 5 days after pollination (dap) following a modified hot borate extraction method (Wan and Wilkins, 1994). The first-strand cDNAs were synthesized with the RETROscript kit (Ambion, Inc., Austin, TX, United States). Quantitative PCR (qPCR) was performed with the SYBR Select Master Mix and the StepOnePlus real-time PCR system (Applied Biosystems, Foster City, CA, United States). The primers used for qPCR are as follows: *InvINH1*, forward 5′-ctgagtgctgctttggatgta-3′, reverse 5′-gttctcgttggtaatcggagac-3′; *InvINH2* forward 5′-aagacccgcaatcgtcatac-3′, reverse 5′-gtcgatgctagggccaaatc-3′; and *Actin2* forward 5′-tccctcagcacattccagcagat-3′, reverse 5′-aacgattcctggacctgcctcatc-3′. *C*_T_ values were normalized against *Actin2*. The use of *Actin2* as the reference gene for qPCR analysis has been previously described (Wang et al., 2010). Most tissues were analyzed with three biological replicates except for silique tissues, which were analyzed with two biological replicates and two technical replicates. The mRNA level of *InvINH1* or *InvINH2* in 3-dap wild-type siliques was set as the reference point (100%) to calculate the relative mRNA levels in other tissues following the -ΔΔ*C*_T_ method (Livak and Schmittgen, 2001).

### Constructs and Plant Transformation

The 5′ flanking region of *InvINH1* or *InvINH2* was cloned into the binary vector pBN-GFP (Wang et al., 2006) to create promoter fusion constructs. In brief, the 5′ flanking regions of *InvINH1* (1172 bp) and *InvINH2* (2238 bp), including the entire 5′ intergenic region and the coding region encoding the first seven amino acids, were amplified from Col-0 genomic DNA using Phusion Polymerase (Thermo Fisher, Waltham, MA, United States). The 5′ UTR region and the first seven amino acids were included in the promoter fusion to ensure proper translation of the GFP gene. Since the N-terminal signal peptide for *InvINH1* and *InvINH2* was predicated to be 20-amino-acid long, the inclusion of the first seven amino acids is unlikely to change the subcellular localization of the GFP protein. The primers used for amplification are as follows: *InvINH1* forward primer (5′-aatgtctagagctgaaatgaaactacatgtgc-3′), *InvINH2* forward primer (5′-cgtttctagacgtctccgattaccaacga-3′), and a common reverse primer (5′-gagaaggatcccaatgaaaccaagaacttcat-3′). The amplified fragments were cloned in frame into the pBN-GFP vector between the XbaI and BamHI sites.

The ectopic expression construct for *InvINH1* was generated by cloning the *ZOU* promoter and *InvINH1* coding region into the binary vector *pBN* (Wang et al., 2006). Since *InvINH1* has no intron, both *InvINH1* coding region and *Zou* promoter were amplified from Col-0 genomic DNA using Phusion Polymerase (Thermo Fisher). The *Zou* promoter region (-2009 bp to +18 bp) was amplified with forward primer (5′-tgattacgccaagcttgtgttacgttgtaacgaattt-3′) and reverse primer (5′-tgctcaccatggatccctcttgagcattagtcatattg-3′), then cloned into *pBN* vector between the HindIII and BamHI site, resulting in construct *pBN-pZOU*. Next, the *InvINH1* coding region (525 bp) was amplified with forward primer (5′-attaggatccatgaagttcttggtttcattggt-3′) and reverse primer (5′-gataggtaccttacaacatattagtaaaagccaaagga-3′), then cloned into *pBN-pZOU* in between the BamHI and KpnI sites, resulting in construct *pBN-pZou-InvINH1*. All constructs were verified by sequencing.

Arabidopsis plant transformation was carried out as described previously (Wang et al., 2006). In brief, *Agrobacterium tumefaciens* strain GV3101 pMP90 (Koncz and Schell, 1986) carrying the appropriate binary vector was used to perform the standard floral dip method (Clough and Bent, 1998). Transgenic seedlings were selected on 0.5x Murashige and Skoog (MS) media containing 35 μg/ml Kanamycin. The presence of the transgene in T1 plants was confirmed using PCR.

### Image Collection and Processing

Seeds were dissected out of the silique as described previously (Wang et al., 2010). To isolate the embryos, seeds were punctured with a dissecting needle and then gently pressed to release the embryos. GFP expression pattern in whole-mount seeds were obtained with a Zeiss LSM 700 inverted confocal microscope (Carl Zeiss, Oberkochen, Germany). Dissected embryos were imaged with Nikon C-DS stereoscopic microscope (Nikon, Tokyo, Japan) equipped with an AxioCam Icc1 digital camera imaging kit (Carl Zeiss). Image processing was performed with Adobe Photoshop CS (Adobe Systems Inc., San Jose, United States).

## Results

### Expression Profiling of Invertases and InvINHs/PMEIs during Seed Development

**FIGURE 1:**
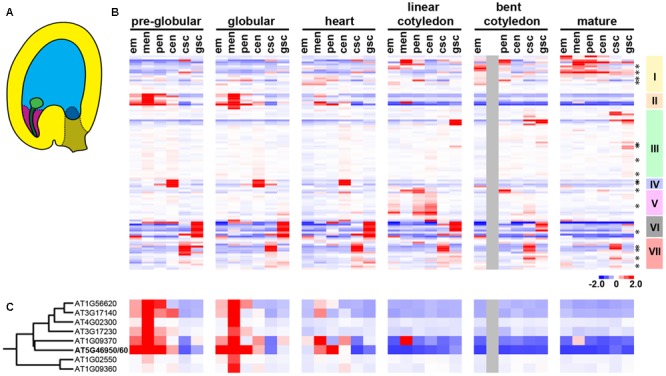
The expression pattern of invertase and members of the InvINH/PMEI superfamily during seed development. **(A)** Illustration depicting a globular stage seed containing embryo proper (light green), suspensor (dark green), chalazal seed coat (brown), general seed coat (yellow), as well as micropylar (purple), peripheral (light blue), and chalazal (dark blue) endosperm. **(B)** Hierarchical clustering of 35 seed samples (in columns) and 107 genes (in rows), including 17 invertases (indicated by asterisks) and 90 InvINH/PMEIs. The early developmental transition in the embryo and the endosperm occurs at the heart stage. Developmental stages are indicated by the stage of embryo development. Each stage includes six seed compartments arranged from left to right as embryo proper (em), micropylar endosperm (men), peripheral endosperm (pen), chalazal endosperm (cen), chalazal seed coat (csc), and general seed coat (gsc). The color represents gene expression level relative to the median level of expression across all samples. Red, high expression; blue, low expression; gray, data not available. Seven clusters of co-regulated genes (I–VII) were identified based on the gene dendrogram. **(C)** A zoomed-in heatmap for Cluster II genes with the data points arranged in the same sequence as **(B)**.

In Arabidopsis, embryo growth rate accelerates after endosperm cellularization (Goldberg et al., 1994; Baud et al., 2008; Hehenberger et al., 2012). To investigate whether the change in embryo growth rate is correlated with any change in invertase activity, we analyzed the temporal and spatial expression pattern of invertase and *InvINHs* by performing hierarchical cluster analysis on a published seed microarray dataset (Belmonte et al., 2013). The dataset includes six developmental time points from pre-globular stage to mature green embryo stage (**Figure 1**). Each stage contains five to six distinct seed compartments captured with laser capture microdissection (**Figure 1**). The list of genes included in our analysis was described in Section “Materials and Methods.” Since it is difficult to distinguish *InvINHs* from *PMEIs* based on sequence conservation alone (Hothorn et al., 2004), we included all members of the *InvINH/PMEI* superfamily in our analysis.

Hierarchical cluster analysis revealed seven co-regulated gene clusters that share distinct spatial and temporal expression patterns during seed developments (**Figure 1**). Some of the co-regulated clusters were specific to seed compartments located at the interface of nutrient transfer, such as chalazal endosperm (**Figure 1B**, Cluster IV), chalazal seed coat (**Figure 1B**, Cluster VII), general seed coat (**Figure 1B**, Cluster VI), and micropylar endosperm (**Figure 1B**, Cluster II). Among these four seed compartments, chalazal endosperm, chalazal seed coat, and general seed coat are located at the seed coat/endosperm interface, suggesting that clusters IV, VI, and VII genes might be involved in nutrient transfer from the maternal tissues to the endosperm during seed development (Li and Berger, 2012). Since we were interested in the nutrient transfer mechanism that regulates embryo growth rate, we focused on Cluster II genes that were specifically expressed in the micropylar endosperm surrounding the embryo (**Figure 1B**). Cluster II contains nine members of the plant *InvINH/PMEI* superfamily represented by eight Affymetrix probes (**Figure 1C**). After the transition at heart-stage (**Figure 1**), cluster II genes were down-regulated in the endosperm (**Figure 1B**), which is expected to cause an increase in invertase or pectin methylesterase activity after endosperm cellularization.

### InvINH1 and InvINH2 Were Specifically Expressed in Reproductive Tissues

To validate the microarray data, two genes from Cluster II were selected for additional expression analysis. These two genes were tentatively named as *InvINH1* (At5g46960) and *InvINH2* (At5g46950). *InvINH1* and *InvINH2* are represented by the same Affymetrix probe due to 93.5% DNA sequence identity between the two genes. Both InvINH1 and InvINH2 proteins are 174 amino acids long and are 88.5% identical to each other. We used three different methods to predict the subcellular location of InvINH1 and InvINH2. The probability of InvINH1 and InvINH2 to be localized to the extracellular space is 75.2, 99.9, and 68% according to PSORT (Nakai and Kanehisa, 1991), Yloc (Briesemeister et al., 2010), and MultiLoc2 (Blum et al., 2009), respectively. The PSORT program predicted that both InvINH1 and InvINH2 have a 20-amino-acid-long N-terminal signal peptide. In addition, InvINH1 has been experimentally confirmed to be a secreted protein associated with the plant cell wall (Irshad et al., 2008), which supports the possibility of InvINH1 to act as an inhibitor of cell-wall-associated enzymes.

**FIGURE 2:**
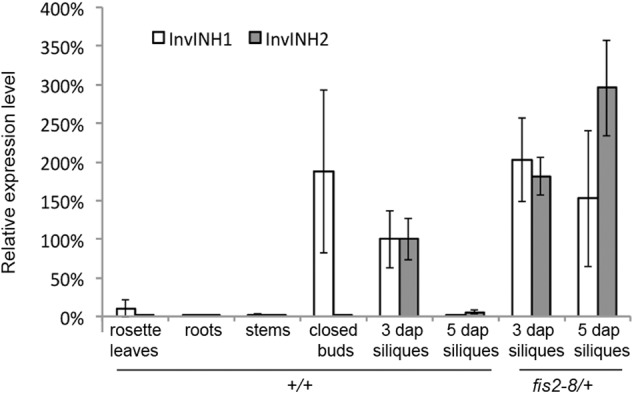
The expression pattern of InvINH1 and InvINH2 in wild type (+/+) and *fis2-8/*+ mutant plants. The mRNA level of InvINH1 and InvINH2 was determined by qRT-PCR in rosette leaves, roots, stems, closed floral buds, and whole siliques at 3 and 5 dap. The expression level in wild-type 3-dap siliques was set as 100%, which was used as a reference point to calculate the relative expression level in other tissues.

To determine whether *InvINH1* and *InvINH2* are specifically expressed in the seed, we used qRT-PCR to analyze the mRNA level of *InvINH1* and *InvINH2* in both vegetative tissues (roots, stems, and rosette leaves) and reproductive tissues (closed floral buds, 3-dap siliques, and 5-dap siliques). Among the reproductive tissues, the 3-dap siliques contained globular-stage embryo and syncytial endosperm, while the 5-dap siliques contained early-torpedo stage embryo and cellularized endosperm. Since *InvINH1* and *InvINH2* are 93.5% identical in DNA sequence, gene-specific qRT-PCR primers were first designed to distinguish *InvINH1* from *InvINH2*. In wild-type plants, the expression level of *InvINH1* and *InvINH2* were dramatically higher in reproductive tissues than in vegetative tissues (**Figure 2**). *InvINH1* was primarily expressed in closed floral buds and 3-dap siliques, while *InvINH2* was primarily expressed in 3-dap siliques (**Figure 2**). Both *InvINH1* and *InvINH2* were strongly down-regulated in 5-dap siliques compared to 3-dap siliques (**Figure 2**). Therefore, both the qRT-PCR and the microarray data suggested that *InvINH1* and *InvINH2* were expressed in the seeds during the syncytial phase and are down-regulated after endosperm cellularization.

In support of our qRT-PCR data, the silique was identified as the structure with the highest expression level for *InvINH1* and *InvINH2* among the 87 anatomical structures annotated by Genevestigator (**Supplementary Figure S1A**). Genevestigator analysis also revealed that the expression of *InvINH1* and *InvINH2* were regulated by additional developmental cues and environmental stimuli (**Supplementary Figure S1B**). For example, *InvINH1* and *InvINH2* were up-regulated during germination (Narsai et al., 2011) and upon *Pseudomonas syringae* inoculation (GEO accession GSE5520, GSE18978). The down-regulation of *InvINH1* and *InvINH2* was observed in the endosperm/seed coat fraction from germinating seeds upon ABA treatment (GEO accession GSE5751), and in seedlings upon exposure to sucrose (Stokes et al., 2013). Moderate expression was also detected in senescent leaves (**Supplementary Figure S1A**). In general, *InvINH1* and *InvINH2* are expressed in tissues that undergo active sugar reallocation, such as the endosperm, germinating seeds, and senescent leaves. Collectively, theses data suggested that *InvINH1* and *InvINH2* are potentially involved in other biological processes in addition to their function during early endosperm development.

### InvINH1 and InvINH2 Were Specifically Expressed in Syncytial Micropylar Endosperm

**FIGURE 3:**
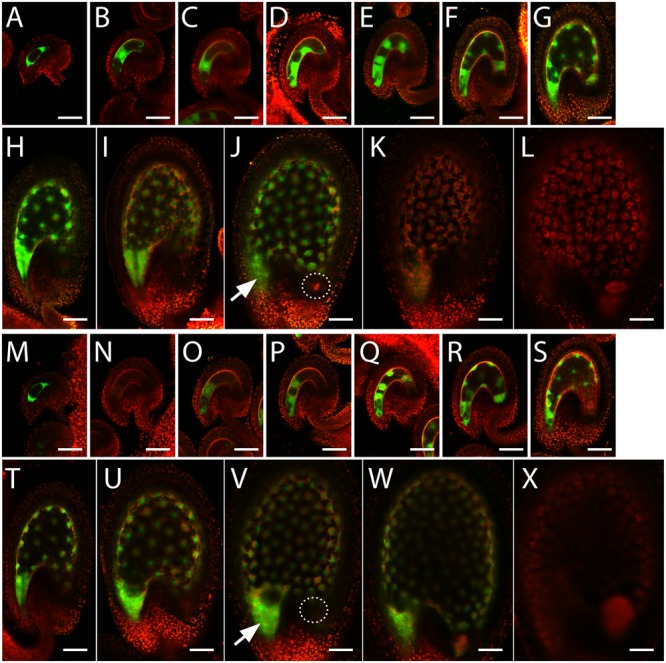
*InvINH1* and *InvINH2* promoter activity in the female gametophyte and the endosperm. Promoter-GFP fusions for *InvINH1* **(A–L)** and *InvINH2* **(M–X)** were analyzed by confocal microscopy in the ovules isolated from stage 12 flowers (Smyth et al., 1990) containing stage FG4 (Christensen et al., 1997) female gametophyte **(A,M)** and from flowers at 1 day after emasculation **(B,N)**. The GFP signals in the fertilized seeds were analyzed at endosperm stage I **(C,O)**, II **(D,P)**, III **(E,Q)**, IV **(F,R)**, V **(G,S)**, VI **(H,T)**, VII **(I,U)**, VIII **(J,V)**, and IX **(K,W)** (Boisnard-Lorig et al., 2001), as well as in seeds containing heart stage embryos **(L,X)**. The embryo stage in **(L,X)** was verified by dissection. GFP (green) and auto-fluorescent signals (red) were imaged simultaneously and merged. All the images were oriented with the micropylar ends on the left and the chalazal ends on the right. Arrow indicates micropylar endosperm. Dashed circle indicates the location of chalazal endosperm. Bar = 50 μm.

To investigate the spatial and temporal expression pattern of *InvINH1* and *InvINH2* during seed development, we generated promoter-GFP fusions for these two genes and analyzed GFP expression pattern in stable transgenic plants. The *InvINH1* promoter-GFP signal was detected in both the female gametophyte and the syncytial endosperm (**Figures 3A–K**). Briefly, the *InvINH1* promoter-GFP activity was detectable in stage FG4 female gametophyte (4-nucleate, Christensen et al., 1997, **Figure 3A**). In the mature female gametophyte at 1 day after emasculation, the promoter activity was only present in the central cell (**Figure 3B**). After fertilization, the promoter-GFP signal was more prevalent in the syncytial endosperm from endosperm stage I (one nucleus) to stage VIII (∼100 nuclei, Boisnard-Lorig et al., 2001, **Figures 3C–J**). After endosperm cellularization at stage IX (**Figure 3K**), the GFP signal decreased dramatically and was no longer detectable in seeds containing heart stage embryos (**Figure 3L**). In seeds that contained globular-stage embryo and syncytial endosperm, the GFP signal was primarily detected in the micropylar endosperm, with weak expression in the periphery endosperm and no expression in the chalazal endosperm (**Figure 3J**). Promoter-GFP expression pattern similar to that of *InvINH1* was also observed for *InvINH2* (**Figures 3M–X**), except that *InvINH2* promoter-GFP signal was not detectable in the female gametophyte at 1 day after emasculation (**Figure 3N**). The observed GFP expression pattern was consistent among the 13 analyzed T1 lines for *pInvINH1-GFP*, as well as among the 11 analyzed T1 lines for *pInvINH2-GFP*. Therefore, both *InvINH1* and *InvINH2* were preferentially expressed in the micropylar endosperm prior to endosperm cellularization, which was in good agreement with the microarray and qRT-PCR data.

### Embryo Growth Was Inhibited by Ectopically Expressed InvINH1

**FIGURE 4:**
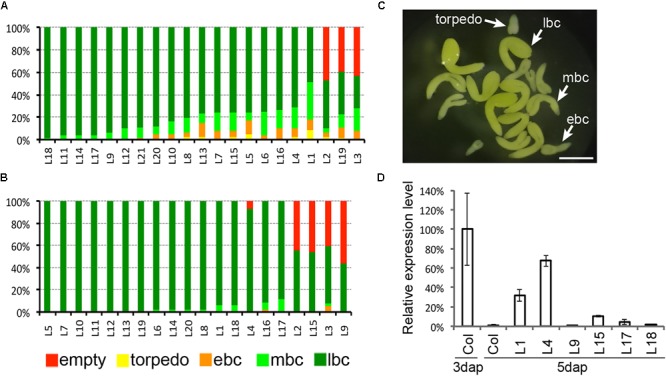
Prolonged expression of *InvINH1* delayed embryo growth. **(A)** The embryo phenotype of 21 individual T1 plants carrying *pZOU-InvINH1 transgene*. **(B)** The embryo phenotype of 20 individual T1 plants carrying *pZou* transgene. **(C)** Dissected embryos from the same silique of a T1 plant carrying *pZOU-InvINH1*. **(D)** Ectopic expression of *InvINH1* in *pZOU-InvINH1* transgenic plants. The *InvINH1* mRNA level in whole siliques was determined by qRT-PCR at 5 dap. The expression level in wild-type (Col-0) siliques at 3 dap was used as a reference point (100%) to calculate the relative expression level in other samples. Around 50–60 seeds were dissected per line to determine the embryo phenotype. empty, undeveloped ovules; ebc, early-bent-cotyledon stage; mbc, mid-bent-cotyledon stage; lbc, late-bent-cotyledon stage (lbc). Bar = 500 μm.

Based on the specific expression pattern of *InvINH1* and *InvINH2*, we hypothesized that their function is to inhibit cell-wall-bound enzymes, such as invertase or pectin methylesterase, that are located at the embryo-endosperm interphase prior to endosperm cellularization. To investigate whether the down-regulation of *InvINH1* after endosperm cellularization is connected to the acceleration in embryo growth rate, we ectopically expressed *InvINH1* after endosperm cellularization using the *ZOU* promoter, which is preferentially active in the micropylar endosperm at both the syncytial stage and the cellularized stage (Yang et al., 2008). Since *InvINH1* and *InvINH2* share similar expression pattern (**Figure 3**) and 93.5% similarity in DNA sequence, there is a possibility that these two genes are functionally redundant. Moreover, it is difficult to generate a double mutant to address the redundancy issue, because these two genes are only 2 kb apart. Therefore, we decided to use the ectopic expression approach to investigate the function of *InvINH1* during seed development. We generated transgenic plants carrying *pZOU-InvINH1* and analyzed seed morphology in 21 independent T1 lines. Embryo morphology was analyzed at the late-bent-cotyledon stage. Six out of the 21 T1 lines had more than 25% delayed embryos within a given silique (**Figure 4A**). Within the same silique from a T1 hemizygous transgenic plant, the normal embryos were at the late-bent-cotyledon stage, while the delayed embryos ranged from early torpedo to mid-bent-cotyledon stage (**Figure 4C**). The delay in embryo growth was transient. At maturation stage, most of the seeds contained normal size embryos. Since the delay in embryo growth was not observed in all the lines, we next investigated if the expression level of the transgene is variable among the T1 lines. We analyzed the mRNA level of *InvINH1* at 5dap in six selected T1 lines that represented different degrees of delayed embryo phenotype (**Figure 4D**). In general, there was a good correlation between the expression level of *InvINH1* and the severity of the phenotype. As the control, we generated and analyzed 20 T1 lines carrying the promoter-only *pZOU* transgene (**Figure 4B**). Seeds containing delayed embryos were observed occasionally. However, none of the *pZOU* lines contained more than 25% delayed seeds (**Figure 4B**). We also observed semi-sterility lines containing close to 50% undeveloped ovules for both *pZou* and *pZou-InvINH1* transgene (**Figures 4A,B**). Semi-sterility is caused by chromosomal rearrangement during T-DNA mediated transformation (Nacry et al., 1998). Since semi-sterility was observed for both *pZou* and *pZou-InvINH1*, this phenotype was not associated with the ectopic expression of *InvINH1*. Collectively, our data indicated that prolonged *InvINH1* expression is sufficient to delay embryo growth.

### InvINH1 and InvINH2 Were Up-Regulated in fis2 Mutant

Since embryo growth was severely suppressed in *mea, fis2, fie*, and *msi1* mutant (Ohad et al., 1996; Chaudhury et al., 1997; Kiyosue et al., 1999; Köhler et al., 2003), we next investigated whether the embryo abortion phenotype in these mutants could be attributed to any changes in *InvINH1* and *InvINH2* expression level. We used qRT-PCR to compare the mRNA level of *InvINH1* and *InvINH2* between the wild-type and *fis2-8* mutant siliques (**Figure 2**). In wild-type plants, *InvINH1* and *InvINH2* were expressed in 3-dap siliques containing syncytial endosperm, and down-regulated in 5-dap siliques containing cellularized endosperm (**Figure 2**). In *fis2* mutant plants, the expression level of *InvINHs1* and *InvINH2* were up-regulated at both 3 and 5 dap (**Figure 2**). More specifically, roughly 2-fold up-regulation was detected at 3 dap, while at least 80-fold up-regulation was detected at 5 dap. The elevation in *InvINH1* and *InvINH2* expression level at 5 dap was not simply the consequence of extended syncytial stage in *fis2* mutant, because elevated expression was detected as early as 3 dap when there was no morphological difference between the wild-type and the mutant seeds. Our data indicated that FIS2-PRC2 complex was required to directly or indirectly silence the expression of *InvINH1* and *InvINH2*, which is expected to increase the activity of cell-wall-bound enzymes, such as invertase or pectin methylesterase after endosperm cellularization.

## Discussion

Proper seed development requires coordinated growth among the embryo, the endosperm, and the seed coat (Garcia et al., 2005; Yang et al., 2008; Ingram, 2010). To determine if embryo growth rate is correlated with invertase activity, we analyzed the spatial and temporal expression pattern of invertases and *InvINH/PMEI*-related genes during Arabidopsis seed development. Our analysis revealed distinct gene clusters that were specifically expressed at the interface between the seed coat and the endosperm, as well as between the endosperm and the embryo. Among these genes, two putative *InvINHs* (*InvINH1* and *InvINH2*) were specifically expressed in the syncytial endosperm surrounding the embryo. After endosperm cellularization, the down-regulation of *InvINH1* and *InvINH2* was dependent on FIS2 function. Moreover, embryo growth was suppressed by the ectopic expression of *InvINH1* in the cellularized micropylar endosperm. Collectively, our data suggested a novel mechanism for the FIS2-PRC2 complex to control embryo growth rate through the repression of *InvINH1* in the micropylar endosperm.

InvINH1 was recently reported as a PMEI (PMEI12) that conferred pathogen resistance by inhibiting PME and strengthening the cell wall during infection (Lionetti et al., 2017). However, this report didn’t directly demonstrate that InvINH1/PMEI12 inhibited pectin methylesterase. InvINHs and PMEIs employ similar scaffold to inhibit two very different enzymes, invertase and pectin methylesterase (Hothorn et al., 2010). However, the conservation in tertiary structure between InvINHs and PMEIs is not reflected in any significant conservation in the primary sequence (Hothorn et al., 2010). Even though several studies have attempted to identify conserved sequence motifs that distinguish InvINHs from PMEIs (Hothorn et al., 2004, 2010; Di Matteo et al., 2005), there are still exemptions to the rule. For example, the PKF motif was suggested as a distinguishing feature for InvINHs (Hothorn et al., 2010). However, this motif is not present in AtC/VIF2, which has been shown to inhibit cell-wall bound invertase *in vitro* (Link et al., 2004). With only a handful of functionally characterized InvINHs and PMEIs, it is still unreliable to distinguish InvINHs from PMEIs based on their primary sequence. Therefore, whether InvINH1 is InvINH or PMEI still remains to be determined by direct enzymatic assay.

InvINH1-mediated suppression of embryo growth may occur via two different mechanisms depending on whether InvINH1 targets invertase or pectin methylesterase. As a pectin methylesterase inhibitor, InvINH1 likely restricts embryo growth via the modification of cell wall composition, since pectin methylesterase is a cell-wall modification enzyme that dimethylesterify cell wall polygalacturonans (Micheli, 2001). As an invertase inhibitor, InvINH1 likely restricts embryo growth by slowing down the flow of sucrose from the endosperm to the embryo before endosperm cellularization. Several lines of evidence suggested that the transport of sucrose from the endosperm to the embryo is important for embryo growth. Based on [^14^C]sucrose tracing experiment, sugar is likely exported as sucrose from the endosperm into the apoplastic space that surrounds the embryo (Morley-Smith et al., 2008). In addition, embryo growth rate was suppressed in mutants that lack functional sucrose transporters in the endosperm and the seed coat (Baud et al., 2005; Chen et al., 2015). Since invertase hydrolyzes sucrose and facilitates sugar transport (Ruan et al., 2010), InvINH1-mediated inhibition of invertase activity could explain the slow embryo growth rate before endosperm cellularization.

The discovery of InvINH1 and InvINH2 provided a missing link between FIS2-PRC2-mediated developmental transition in the endosperm and the accelerated embryo growth that follows. Several attempts have been made to identify the genes targeted by FIS2-PRC2 complex during endosperm cellularization (Tiwari et al., 2010; Weinhofer et al., 2010). However, it has been difficult to tease out the syncytial program from other developmental programs that are suppressed by the FIS2-PRC2 complex, such as the flowering and embryonic programs (Makarevich et al., 2006; Weinhofer et al., 2010). *InvINH1* is the first structural gene from the collection of known FIS2-PRC2 targets that might offer an explanation why embryo growth during syncytial endosperm phase is limited. In addition to being up-regulated in *fis* mutants such as *mea* and *fis2*, InvINH1 and InvINH2 were also up-regulated in interploidy crosses with excess paternal genome (Erilova et al., 2009; Tiwari et al., 2010). Since paternal-excess cross leads to prolonged syncytial stage (Scott et al., 1998), both these studies and our data suggested that InvINH1 and InvINH2 are specifically associated with the syncytial endosperm program. Furthermore, our data suggested that the expression of InvINH1 during the syncytial stage may be connected to the slow embryo growth rate observed before endosperm cellularization (Baud et al., 2008).

It remains to be determined whether *InvINH1* and *InvINH2* are direct or indirect targets of the FIS2-PRC2 complex. The FIS2-PRC2 complex maintains gene silencing and genomic imprinting of several endosperm-expressed genes through the methylation of H3K27 (Köhler et al., 2005; Baroux et al., 2006; Fitz Gerald et al., 2009). However, *InvINH1* and *InvINH2* have not been identified as imprinted genes (Gehring et al., 2011; Hsieh et al., 2011; McKeown et al., 2011; Wolff et al., 2011), nor have they displayed significant enrichment of methylated H3K27 (Weinhofer et al., 2010). Therefore, *InvINH1* and *InvINH2* may not be directly targeted by FIS2-PRC2 complex. Instead, additional regulators may exist to connect *InvINH1* and *InvINH2* to the FIS2-PRC2 regulatory network. Future studies aimed at identifying the upstream regulators of *InvINH1* and *InvINH2* will provide a more definitive answer to this question.

## Author Contributions

DW designed research and wrote the paper. DW, BZ, and MD performed research and analyzed data.

## Conflict of Interest Statement

The authors declare that the research was conducted in the absence of any commercial or financial relationships that could be construed as a potential conflict of interest. The reviewer T-FH and handling Editor declared their shared affiliation.

## Footnotes

1 http://bioconductor.org/packages/2.0/bioc/html/gcrma.html

2 http://www.cazy.org

3 http://supfam.org/SUPERFAMILY

4 http://psort.hgc.jp/form.html

5 http://abi.inf.uni-tuebingen.de/Services/MultiLoc2

6 http://abi.inf.uni-tuebingen.de/Services/Yloc/webloc.cgi

7 https://genevestigator.com/gv/

## Supplementary Material

FIGURE S1 *In silico* expression analysis of *InvINH1* and *InvINH2*. **(A)** The relative expression level was illustrated as a heatmap containing 87 anatomical features that were hierarchically organized by the Anatomy tool from Genevestigator. **(B)** The Perturbations tool from Genevestigator was used to generate the heatmap displaying the experimental stimuli that induced greater than 1.5-fold changes in the expression level of *InvINH1* and *InvINH2*. *InvINH1* and *InvINH2* are represented by the same Affymetrix probe, 248823_s_at.
