# Supplementary figures and images for: Prolonged Expression of a Putative Invertase Inhibitor in Micropylar Endosperm Suppressed Embryo Growth in Arabidopsis
Source: Front Plant Sci. 2018 Jan 30;9:61. doi: 10.3389/fpls.2018.00061 (PMC5797552; doi:10.3389/fpls.2018.00061)

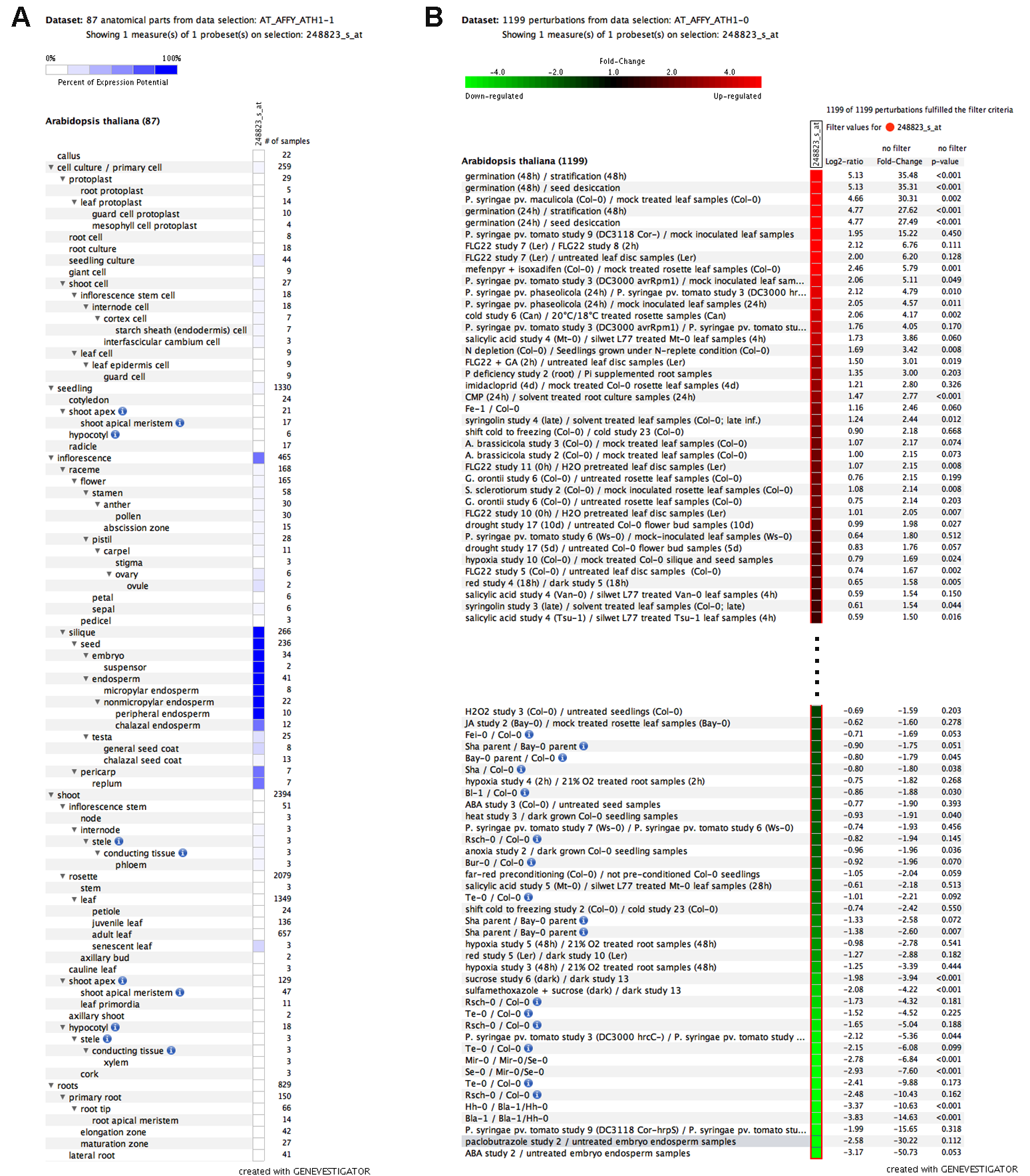

Supplement: FIGURE S1 — In silico expression analysis of InvINH1 and InvINH2. (A) The relative expression level was illustrated as a heatmap containing 87 anatomical features that were hierarchically organized by the Anatomy tool from Genevestigator. (B) The Perturbations tool from Genevestigator was used to generate the heatmap displaying the experimental stimuli that induced greater than 1.5-fold changes in the expression level of InvINH1 and InvINH2. InvINH1 and InvINH2 are represented by the same Affymetrix probe, 248823_s_at. [file Image_1.TIF]
